# Optical Coherence Tomography Angiography to Estimate Early Retinal Blood Flow Changes after Uncomplicated Cataract Surgery

**DOI:** 10.3390/vision6030038

**Published:** 2022-06-24

**Authors:** Antonio Baldascino, Matteo Ripa, Matteo Mario Carlà, Tomaso Caporossi, Giulia Grieco, Gloria Gambini, Umberto De Vico, Giuseppe Raguso, Raphael Kilian, Clara Rizzo, Stanislao Rizzo

**Affiliations:** 1 Ophthalmology Unit, Fondazione Policlinico Universitario A. Gemelli IRCCS, 00168 Rome, Italy; antonio.baldascino@policlinicogemelli.it (A.B.); mm.carla94@gmail.com (M.M.C.); tomaso.caporossi@gmail.com (T.C.); giuliagrieco.md@gmail.com (G.G.); gambini.gloria@gmail.com (G.G.); umbertodevico@gmail.com (U.D.V.); graguso12494@gmail.com (G.R.); stanislao.rizzo@policlinicogemelli.it (S.R.); 2 Ophthalmology Unit, Catholic University “Sacro Cuore”, 00168 Rome, Italy; 3 Ophthalmology Unit, University of Verona, 37129 Verona, Italy; raphaelkilian8@yahoo.it; 4 Ophthalmology Unit, Department of Surgical, Medical, Molecular and Critical Area Pathology, University of Pisa, 56124 Pisa, Italy; clararizzo2@gmail.com; 5 Consiglio Nazionale delle Ricerche, Istituto di Neuroscienze, 56127 Pisa, Italy

**Keywords:** cataract surgery, ophthalmic surgery, vessel density, foveal avascular zone, optical coherence tomography angiography

## Abstract

Background: To investigate macular microvascular changes after uncomplicated phacoemulsification surgery according to the cataract severity grade. Methods: Retrospective, cross-sectional study involving 23 eyes of 23 patients who underwent elective cataract extraction. All patients underwent routine ophthalmologic examination, including optical coherence tomography angiography (OCTA) at baseline (preoperative visit, T0) and seven days postoperatively (T7). OCTA scans were obtained with the spectral domain system Cirrus 5000 (Carl Zeiss Meditec, Inc., Dublin, CA, USA), and 3 mm × 3 mm raster fovea-centered scans were obtained to evaluate the superficial capillary plexus (SCP) vessel density, perfusion density, and foveal avascular zone (FAZ) parameters. Results: SCP perfusion density significantly increased from 28.3 ± 5.73% to 33.74 ± 4.13% after the surgery (*p* < 0.001). Similarly, SCP vessel density significantly increased from 15.14 ± 3.41 mm^−1^ to 18.14 ± 2.57 mm^−1^ after surgery (*p* < 0.001). The mean preoperative FAZ area significantly increased from 0.27 ± 0.12 mm to 0.24 ± 0.11 mm seven days postoperatively (*p* = 0.008). When comparing softer and harder cataracts, no significant variations in SCP vessel density, as well as SCP perfusion density parameters and the FAZ area, perimeter, and circularity index, were noted before and after surgery. Conclusions: Macular SPC vessel density and macular SCP perfusion density increase after uncomplicated cataract surgery regardless of the cataract severity.

## 1. Introduction

Cataract remains the leading cause of blindness in middle-income and low-income countries, with an estimated 16 million people worldwide [1,2]. Today, phacoemulsification cataract surgery is one of the most common anterior segment surgical procedures. Reports evidenced that ocular blood flow may improve after cataract surgery, even if post-operative effects on the retinal vascular system are still not clear [3].

As demonstrated by Zhao et al., cataract surgery induces a significant increase in vessel density at the parafoveal and perifovea regions, along with a decrease in the foveal avascular zone (FAZ) [4].

Various methods such as Doppler ultrasound and fundus fluorescein angiography (FFA) can observe retinal blood flow. However, these clinical applications have some limitations, as Doppler ultrasound can only see large vessels with low resolution, and FFA is invasive and difficult to quantify [5]. Optical coherence tomography angiography (OCTA) may produce images of blood flow, offering an unprecedented resolution of all retinal vascular layers in a rapid, non-invasive fashion.

OCTA has been used to assess the FAZ area and macular vascular flow density in healthy and pathologic eyes [6]. Starting from this assumption, this study aimed to describe and assess the vasculature changes in terms of perfusion density as well as vessel density and FAZ parameters (Area, Circularity Index, and Perimeter) in the macular area after uncomplicated phacoemulsification in patients with neither ocular nor systemic diseases. Furthermore, we aimed to evaluate whether the stage of cataract evolution may influence post-operative OCTA parameters.

## 2. Materials and Methods

### 2.1. Patients

In this retrospective cross-sectional study, we collected pre-and post-operative data from 23 people who underwent elective cataract extraction performed between 1 June 2020 and 1 November 2020 at our ophthalmology unit (Fondazione Policlinico Universitario A. Gemelli IRCCS, Rome, Italy). Data from patients who underwent phacoemulsification surgery with intraocular lens (IOL) implantation were retrieved and collected.

This study was approved by the Institutional Review Board and followed the tenets of the Declaration of Helsinki. 

The inclusion criteria for the study were the presence of a nuclear or cortical cataract with no concomitant intraocular disease and intraocular pressure (IOP) lower than 21 mmHg. Eyes with IOP higher than 21 mmHg, with a diagnosis of posterior subscapular or posterior polar cataract, history of ocular trauma or intraocular surgery, axial length > 26, or any abnormal intraocular findings, were excluded. 

Patients diagnosed with glaucoma or any retinopathies that might result in the abnormal microvascular network (e.g., age-related macular degeneration, diabetic retinopathy, retinal vascular disorders, etc.) or previously treated by laser or photodynamic therapy were also excluded. In addition, patients with either a previous or current history of systemic diseases such as diabetes and inflammatory or cardiovascular diseases that may interfere with retinal vascular abnormalities were excluded. Furthermore, poor OCT images due to severe cataracts, unstable fixation, or any signs of intraoperative or postoperative complications were also grounds for exclusion.

### 2.2. Examinations

All patients underwent routine examinations prior to cataract surgery, including the measurement of corrected distance visual acuity (CDVA) in the logarithm of the minimum angle of resolution (logMAR), the measurement of intraocular pressure (IOP) with a non-contact tonometer, the measurement of axial length (AL) with partial coherence interferometry (IOLMaster 700, Carl Zeiss-Meditec AG, Jena, Germany), a slit lamp biomicroscopy, cataract grading with the Lens Opacities Classification System III [7], and a dilated fundus evaluation using either 78D or 90D lenses. 

After pupil dilation, obtained with 1% tropicamide solution, enrolled eyes underwent OCTA scans, at baseline (preoperative visit, T0) and after surgery, seven days postoperatively (T7). To be included in the analysis, scans had to show signal strength scores greater than 6 to allow quantitative assessment. In addition, the peripheral retinal vascular pattern, as well as the potential peripheral neovascularization, was visually assessed at baseline and one week postoperatively using a confocal device. (Eidon, Centervue, Padova, Italy)

Furthermore, in a post hoc analysis, we divided our sample in two groups according to the cataract severity grade:

1. Group 1: Patients affected by less severe cataracts (nuclear opalescence (NO), nuclear color (NC), cortical cataract (C) ≤ 3).
2. Group 2: Patients affected by severe cataracts (nuclear opalescence (NO), nuclear color (NC), cortical cataract (C) > 3).

### 2.3. Image Acquisition

Optical coherence tomography angiography scans were obtained with the spectral domain system Cirrus HD-OCT 5000 along with AngioPlex software (Carl Zeiss Meditec, Inc., Dublin, CA, USA), and 3 mm × 3 mm raster scans centered on the fovea were obtained. 

Using an 840 nm center wavelength and 68,000 A-scans per second, AngioPlex creates high-resolution microvascular images of the retina and choroid, providing quantitative measurements of the superficial capillary plexus’s (SCP) vessel density (VD), perfusion density (PD), and foveal avascular zone (FAZ).

The en-face images of the SCP were captured using the customized segmentation between an inner boundary at the inner limiting membrane (ILM) and an outer boundary at the inner plexiform layer (IPL), whereas the DCP was visualized between the IPL and the outer plexiform layer (OPL).

The macula was segmented into two concentric circles with 1 mm and 3 mm diameters. The inner 1 mm diameter circle was defined as the “inner ring”, the 3 mm annular ring was defined as the “central ring”, and the whole ETDRS 3 mm × 3 mm ring was henceforth defined as the “whole or full ring”. The Cirrus AngioPlex OCTA device (Zeiss, Dublin, CA, USA) automatically measures perfusion density (PD) and vessel density (VD). PD is defined as the percentage of an area with circulation, and VD is the length of vessels with circulation in a defined area. SCP’s PD and VD were measured automatically by the OMAG algorithm used in the AngioPlex software in the central, inner, and full ring. However, the DCP’s PD and VD were not measured. The AngioPlex software automatically measured the (FAZ) area of the superficial capillary plexus. The FAZ area was defined as the segmented FAZ region’s size, whereas the FAZ contour’s length determined the perimeter.

OCT via the AngioPlex Metrix toolbox identifies the FAZ border from the en-face superficial retinal layer images and applies a yellow overlay to the FAZ. Then, the area, perimeter, and circularity of the FAZ are then shown in tabular form. The area (mm^2^), perimeter (mm), and circularity index (%) of FAZ measurements were reported.

Two OCT specialists (A.B. and T.C.) blinded to the subject’s cataract severity grade examined all SCP and DCP images according to a pre-specified protocol. If consensus could not be reached, a third specialist (S.R.) was consulted for the final decision. The protocol evaluated either the presence or the absence of five parameters: motion (image interruptions, shifts, discontinuities, or vascular ghosting due to ocular movements that may increase the vessel density), decentration (center of scan not aligned with the center of the macula, either increasing or decreasing the vessel density), defocus (reduced retinal reflectivity due to poor autofocus), masking (light blockage due to anterior or posterior ocular abnormalities such as vitreous opacities and pigment clumps, which do not permit the beam to reach deeper layers), and segmentation errors (unclear boundary of the different layers of the retina). Images with at least one artifact were discarded.

### 2.4. Surgical Technique

All cataract surgeries were performed by an expert surgeon (A.B.) using the Centurion Vision System (Alcon Laboratories, Inc., Fort Worth, TX, USA).

After topical anesthesia, a 2.2 mm clear corneal self-sealing incision, continuous capsulorhexis, hydrodissection, phacoemulsification, and irrigation/aspiration of the residual lens cortex were sequentially performed. A foldable IOL (Alcon AcrySof^®^ single-piece SA60WT, Alcon Laboratories Inc.) was implanted in the capsular bag. The effective phacoemulsification time (in seconds) and phacoemulsification cumulative dissipated energy (CDE) (expressed in Joules, J) of the phacoemulsification machine were documented.

Postoperative treatment consisted of: Dexamethasone 0.1% and Netilmicin 0.3% eyedrops administered four times a day for seven days and then tapered every seven days for one month; Levofloxacin 0.5% eyedrops administered four times for ten days; Diclofenac sodium 0.1% eyedrops administered four times a day for one month.

### 2.5. Statistical Analysis

All data were collected using the REDCap platform [8], and all statistical analyses were performed using the 27th version of SPSS (IBM-SPSS, Chicago, IL, USA).

Descriptive statistics techniques summarized all variables included in the study. In-depth, qualitative variables were expressed as the absolute and percentage frequency. As for quantitative variables, we performed the Shapiro—Wilk test to assess their distribution.

In addition, we further evaluate skewness and kurtosis by running a z-test. Then, if they were normally distributed, they were described as mean and standard deviation (SD), otherwise they were described as median and interquartile range (IQR).

Between-group differences for each parameter considered (age, sex, mean correct distance visual acuity (CDVA), effective phacoemulsification time (EPT), phacoemulsification energy, and postoperative follow-up examination interval) were assessed, as for qualitative variables, either by the Fisher Exact test or the Chi-square test, with Yates’ correction as appropriate. Quantitative variables were evaluated by Student t-test if normally distributed; otherwise, the Mann—Whitney U test was applied. Specifically, “Intra-group” comparative analyses, based on preoperative and postoperative quantitative variables, were conducted on both groups. Specifically, the pre-and post-intervention measures of quantitative variables were compared separately for each group using Student’s *t*-test for paired samples. The effect of cataract severity on the mean increase difference [Δ (T7-T1)] between the preoperative and postoperative SCP perfusion density, vessel density, and FAZ parameters in patients with different cataract severity grades was assessed by linear regression analysis. A *p*-value < 0.05 was considered statistically significant.

## 3. Results

### 3.1. Demographics and Main Clinical Data

Twenty-three eyes of twenty-three patients with senile cataracts fulfilled the assessment visits at baseline and after surgery. All patients did not develop any surgical complications during and after surgery.

The mean correct distance visual acuity (CDVA) before surgery was 0.55 ± 0.23 LogMar (range 0.10–1.00), whereas the mean CDVA after surgery was 0.07 ± 0.08 (range 0.00–0.2). Therefore, the CDVA was significantly improved (*p* < 0.01).

The patients’ mean age was 75.86 ± 8.85 years (range 55–98 years), the mean effective phacoemulsification time was 91.56 + 25.56 s, and the mean phacoemulsification energy was 11.83 + 5.05 µJ. The mean interval of the postoperative follow-up examination was 7.70 ± 0.97 days.

### 3.2. Microvasculature Parameters

All parameters of perfusion density and vessel density significantly increased after the cataract surgery when comparing baseline (T0) to 1-week follow-up (T7). (*p* < 0.05).

The preoperative SCP vessel density in the central ring was 8.20 ± 3.30 mm^−1^ at T0, increasing to 11.24 ± 4.84 mm^−1^ at T7 (*p* = 0.001). SCP vessel density in the inner ring was 16.04 ± 3.62 mm^−1^ at baseline, and 19.02 ± 2.64 mm^−1^ at T7, showing a statistically significant increase compared to baseline (*p* < 0.001). At last, SCP vessel density in the full ring increased from T0 to T7, with a significant variation when comparing baseline to 1-week follow-up (15.14 ± 3.41 vs. 18.14 ± 2.57 mm^−1^, *p* < 0.001).

The preoperative SCP perfusion density in the central ring, inner ring, and whole area was 14.95 ± 6.05%, 29.97 ± 6.18%, and 28.3 ± 5.73%, respectively. A significant variation was found at T7 follow-up for all these parameters: 19.98 ± 8.93% for the central ring (*p* = 0.003), 35.59 ± 4.17% for the inner ring (*p* = 0.001), and 33.74 ± 4.13% for the full ring (*p* < 0.001) (Table 1 and Table 2).

The FAZ parameters decreased one week after surgery. Notably, the mean preoperative FAZ area significantly decreased from 0.27 ± 0.12 mm^2^ to 0.24 ± 0.11 mm^2^ seven days after surgery (*p* = 0.008). The mean preoperative FAZ Perimeter decreased from 2.31 ± 0.54 mm to 2.17 ± 0.58 mm after the surgery in a non-significant way (*p* = 0.057), and the mean preoperative circularity index also did not show any statistical variation at T7 (0.59± 0.05 to 0.62 ± 0.09, *p* = 0.49) (Table 3).

### 3.3. Subgroup Analysis Related to Cataract Severity

Fourteen eyes belonged to group 1 (less severe cataracts), whereas nine eyes belonged to group 2 (severe cataracts). Group 1’s mean CDVA before surgery was 0.44 ± 0.10 LogMar (range 0.3–0.7), whereas the mean CDVA after surgery was 0.03 ± 0.06 (range 0.00–0.2).

Group 2’s mean CDVA before surgery was 0.71 ± 0.29 LogMar (range 0.1–1), whereas the mean CDVA after surgery was 0.12 ± 0.08 (range 0.00–0.2).

Group 1’s phacoemulsification cumulative dissipated energy (CDE) (expressed in Joules, J) of the phacoemulsification was 8.70 ± 2.13 µJ, whereas Group 2’s CDE was 16.71 ± 4.35 µJ. Group 1’s effective phacoemulsification time (in seconds) was 75.60 ± 7.8 s, whereas group 2’s effective phacoemulsification time was 116.38 ± 23.59 s.

### 3.4. Microvasculature Parameters Related to Cataract Severity

**Figure 1:**
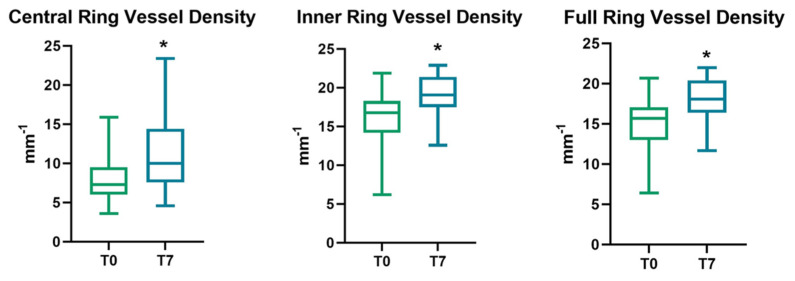
Evolution of SCP vessel density parameters (expressed as the mean ± SD) at follow-up, with an asterisk to indicate a significant variation. SCP = superficial capillary plexus; T0 = baseline values; T7 = 1-week follow-up.

**Figure 2:**
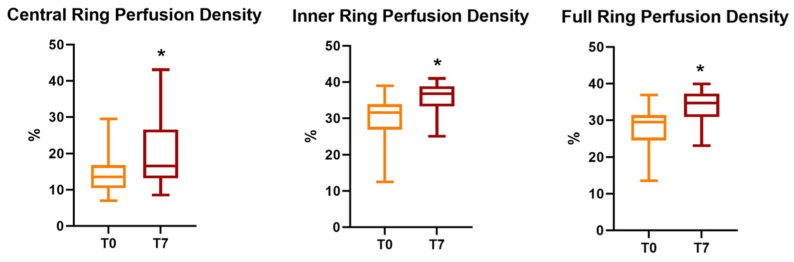
Evolution of SCP perfusion density parameters (expressed as the mean ± SD) at follow-up, with an asterisk to indicate a significant variation. SCP = superficial capillary plexus; T0 = baseline values; T7 = 1-week follow-up.

All parameters of perfusion density and vessel density significantly increased after the cataract surgery, regardless of cataract severity. (*p* < 0.05) (Figure 1 and Figure 2).

Group 1’s mean preoperative SCP perfusion density in the central ring, inner ring, and full ring was 14.87 ± 6.33%, 31.20 ± 4.98%, and 29.35 ± 5.01%, respectively, which significantly increased to 18.22 ± 7.11%, 35.89 ± 4.38%, and 33.75 ± 4.61% after the surgery (*p* = 0.03, *p* = 0.007, and *p* = 0.005, respectively).

Group 2’s mean preoperative SCP perfusion density in the central ring, inner ring, and full ring was 15.06 ± 5.96%, 28.06 ± 7.62%, and 26.66 ± 6.68%, respectively, which significantly increased to 22.72 ± 11.11%, 35.13 ± 4.04%, and 33.73 ± 3.52% after the surgery (*p* = 0.039, *p* = 0.006, and *p* = 0.005, respectively).

There was no significant difference between the mean preoperative and postoperative SCP perfusion density in the central ring, inner ring, and full ring between groups (*p* = 0.52, *p* = 0.24, *p* = 0.28, and *p* = 0.24, *p* = 0.88, and *p* = 0.99, respectively). In addition, there was no significant mean increase difference [Δ (T7-T1)] between the preoperative and postoperative SCP perfusion density in the central ring, inner ring, and full ring between groups (*p* = 0.17, *p* = 0.32, *p* = 0.25, respectively). Linear regression was calculated to predict the mean increase difference [Δ (T7-T1)] between the preoperative and postoperative SCP perfusion density in the central ring, inner ring, and full ring in both groups from the cataract severity grade. A non-significant linear regression was found (F = 2.02, *p* = 0.17, R^2^ = 0.06; F = 1.03, *p* = 0.32, R^2^ = 0.47; F = 1.37, *p* = 0.25, R^2^ = 0.06).

Group 1’s mean preoperative SCP vessel density in the central ring, inner ring, and full ring was 8.17 ± 3.34 mm^−1^, 16.68 ± 3.04 mm^−1^, and 15.70 ± 3.02 mm^−1^, respectively, which significantly increased to 10.27 ± 3.94 mm^−1^, 19.27 ± 2.68 mm^−1^, and 18.25 ± 2.93 mm^−1^ after the surgery (*p* = 0.016, *p* = 0.001, and *p* = 0.01, respectively). 

Group 2’s mean preoperative SCP vessel density in the central ring, inner ring, and full ring was 8.25 ± 3.25 mm^−1^, 15.04 ± 4.37 mm^−1^, and 14.26 ± 3.96 mm^−1^, respectively, which significantly increased to 12.74 ± 5.89 mm^−1^, 18.63 ± 2.36 mm^−1^, and 17.97 ± 2.93 mm^−1^ after the surgery (*p* = 0.022, *p* = 0.008, and *p* = 0.006, respectively).

There was no significant difference between the mean preoperative and postoperative SCP vessel density in the central ring, inner ring, and full ring between groups (*p* = 0.95, *p* = 0.22, *p* = 0.98, and *p* = 0.24, *p* = 0.58, and *p* = 0.60, respectively). In addition, there was no significant mean increase difference [Δ (T7-T1)] between the mean preoperative and postoperative SCP vessel density in the central ring, inner ring, and full ring between groups (*p* = 0.14, *p* = 0.48, and *p* = 0.39, respectively). Linear regression was calculated to predict the mean increase difference [Δ (T7-T1)] between the preoperative and postoperative SCP vessel density in the central ring, inner ring, and full ring in both groups from the cataract severity grade. A non-significant linear regression was found (F = 2.27, *p* = 0.14, R^2^ = 0.05; F = 0.51, *p* = 0.48, R^2^ = 0.02; F = 0.75, *p* = 0.39, R^2^ = 0.01).

The FAZ decreased significantly seven days after surgery in both groups. The mean preoperative FAZ area in group 1 was 0.27 ± 0.12 mm^2^, which decreased to 0.25 ± 0.10 mm^2^ after the surgery in a non-significant way (*p* = 0.05). The mean preoperative FAZ area in group 2 was 0.25 ± 0.14 mm^2^, which decreased to 0.22 ± 0.12 mm^2^ after the surgery in a non-significant way (*p* = 0.08). There was no significant difference between the mean preoperative and postoperative FAZ area between the groups (*p* = 0.72 and *p* = 0.48, respectively). In addition, there was no significant mean increase difference [Δ (T7-T1)] between the mean preoperative and postoperative FAZ area between groups (*p* = 0.5).

The mean preoperative FAZ perimeter in group 1 was 2.35 ± 0.49 mm, which decreased to 2.23 ± 0.57 mm after the surgery in a non-significant way (*p* = 0.23). The mean preoperative FAZ perimeter in group 2 was 2.25 ± 0.63 mm, which decreased to 2.06 ± 0.61 mm after the surgery) in a non-significant way (*p* = 0.14). There was no significant difference between the mean preoperative and postoperative FAZ perimeter between groups (*p* = 0.68 and *p* = 0.51, respectively). In addition, there was no significant mean increase difference [Δ (T7-T1)] between the mean preoperative and postoperative FAZ perimeter between groups (*p* = 0.65).

The mean preoperative circularity index in group 1 was 0.59 ± 0.06, which increased to 0.62 ± 0.09 after the surgery in a non-significant way (*p* = 0.05). The mean preoperative circularity index in group 2 was 0.60 ± 0.03, which remained stable after the surgery (0.60 ± 0.09) in a non-significant way (*p* = 0.9). There was no significant difference between the mean preoperative and postoperative FAZ circularity index between groups (*p* = 0.97 and *p* = 0.61, respectively). In addition, there was no significant mean increase difference [Δ (T7-T1)] between the mean preoperative and postoperative FAZ circularity index between groups (*p* = 0.66). 

A linear regression was calculated to predict the mean increase difference [Δ (T7-T1)] between the preoperative and postoperative FAZ area, perimeter, and circularity index in both groups from the cataract severity grade. A non-significant linear regression was found (F = 0.46, *p* = 0.5, R^2^ = 0.02; F = 0.20, *p* = 0.65, R^2^ = 0.03; F = 0.19, *p* = 0.66, R^2^ = 0.03) (Table 4 and Table 5).

## 4. Discussion

Nowadays, OCTA is a reliable noninvasive tool that evaluates the retinal vascular pattern and circulation. OCTA is a reliable tool for detecting these parameters with a high degree of repeatability [9]. Many studies indeed assessed OCTA’s utility for ocular pathology monitoring, thanks to the ease of use, the short acquisition times, and non-invasiveness [10]. The FAZ area, perimeter, and circularity index are the most common parameters related to macular retinal vasculature studied by OCTA, which is capable of producing highly reproducible FAZ images and has been shown to identify the changes in the perifoveal capillary circulation [11,12], which seemed to differ in terms of oxygen saturation and blood flow velocity when compared to the foveal area [13].

In our study, we aimed to evaluate the retinal vasculature changes in terms of perfusion density as well as vessel density at the macular area after uncomplicated phacoemulsification in patients with neither ocular nor systemic diseases. Our findings are consistent with those reported in previous research held by Zhao et al. and Yu et al. [4,14].

Zhao et al. assessed that the increased vascular density was related to three factors: (1) increased pulsatile ocular blood flow after cataract surgery due to the postoperative decrease in IOP (a drop in IOP determines an increased pulsatile ocular blood flow after surgery, leading to higher perfusion of the smaller retinal vessel) [15]; (2) an enhanced postoperative inflammation (expression of proinflammatory genes and proteins. These cytokines were reported to cause vessel dilation and a breakdown of the blood-retinal barrier) [16]; (3) an increase in light exposure (increased light exposure could lead to increased activity and more metabolic demands in the retina, even if this topic is still unclear). In contrast, Yu et al. assessed that increased perfusion and vessel density were not related to the increased inflammatory status after surgery, which could lead to increased perfusion, but strictly related to improved visibility after lens extraction [14]. Indeed, given the numerical decrease in artifacts, the significant improvement in vessel continuity, the increase in signal strength, and the substantial change in quantitative parameters, they believed that lower density values before surgery were associated with lower image quality due to media opacities.

To further investigate whether the lens density and thus the lower quality image may be related to the VD and PD’s increase after lens extraction, we split our sample into two subgroups according to lens opacity grade.

Analyzing the mean increase difference [Δ (T7-T1)] between the preoperative and postoperative SCP perfusion density and vessel density, we did not find any substantial variations between the two groups (group 1: soft cataracts; group 2: hard cataracts) (*p* > 0.05). Therefore, we can assess that cataract severity does not significantly affect OCTA-calculated parameters. In addition, as the baseline (T0) VD and PD values were quite similar between the two groups (*p* > 0.05), the media opacity density (lens density) did not significantly alter the PD and VD.

To further establish this hypothesis, we run a linear regression to evaluate if the cataract severity could be related to the mean increase difference [Δ (T7-T1)] between the preoperative and postoperative SCP perfusion density, vessel density, and FAZ parameters in both groups. It showed us that the lens density does not statistically significantly predict the mean increase difference [Δ (T7-T1)] between the preoperative and postoperative SCP perfusion density, vessel density, and FAZ parameters in both groups. (*p* > 0.05) 

Thus, in our small sample, the lens density impact on image quality in terms of artifacts and signal strength did not affect the mean increase of VD and PD in the higher density group. These findings show that the enhanced retinal vascular density and perfusion along with the FAZ area reduction are not strictly related to the image quality.

However, this study has several limitations. First, the results may not be generalizable due to the limited sample size. Second, we did not deeply analyze the peripheral retinal vascular abnormalities. Third, the increased macular retinal vessel density and perfusion were assessed just one week after surgery when the postoperative inflammation is in its thriving phase. Finally, we included only patients with neither systemic nor ocular disease.

Therefore, the assessment of confounding factors such as medications, medication adherence, early-stage diseases, cardiovascular diseases, or inflammatory disorders that may be related to vessel changes could not be assessed in the study’s population. Therefore, further investigations with a longer follow-up should be considered due to the known links between diabetes, cataract, and blood vessel changes. In addition, evaluating the underlying mechanisms will be of great importance and interest.

In conclusion, after uncomplicated lens extraction, the macular perfusion density and the vessel density significantly increased regardless of the cataract severity grade. Studies with a bigger sample size are required to investigate the underlying mechanisms further.

## Figures and Tables

**Table 1:** Vessel density changes in the SCP after cataract surgery. Abbreviations: SCP = superficial capillary plexus; mm= millimeter; T0 = baseline values; T7 = 1-week follow-up, * = statistically significant.

Area	Vessel Density (mm^−1^)		
	T0	T7	*p*-Value
Central	8.20 ± 3.30	11.24 ± 4.84	0.001 *
Inner	16.04 ± 3.62	19.02 ± 2.64	<0.001 *
Full	15.14 ± 3.41	19.02 ± 2.64	<0.001 *

**Table 2:** Vessel perfusion changes in the SCP after cataract surgery. Abbreviations: SCP = superficial capillary plexus; T0 = baseline values; T7 = 1-week follow-up, * = statistically significant.

Area	Perfusion Density (%)		
	T0	T7	*p*-Value
Central	14.95 ± 6.05	19.98 ± 8.93	0.003 *
Inner	29.97 ± 6.18	35.59 ± 4.17	0.001 *
Full	28.3 ± 5.73	33.74 ± 4.13	<0.001 *

**Table 3:** Foveal avascular zone (FAZ) area, perimeter, and circularity index changes after cataract surgery.

FAZ	T0	T7	*p*-Value
Area (mm^2^)	0.27 ± 0.12	0.24 ± 0.11	0.008 *
Perimeter (mm)	2.31 ± 0.54	2.17 ± 0.58	0.057
Circularity Index	0.59 ± 0.05	0.62 ± 0.09	0.49

Abbreviations: FAZ = Foveal avascular zone area; mm = millimeter; T0 = baseline values; T7 = 1-week follow-up, * = statistically significant.

**Table 4:** Subgroup analysis of perfusion density, vessel density, and the FAZ area, perimeter, and circularity index changes after cataract surgery according to different stages of cataract severity. FAZ = foveal avascular zone.

		Group 1 (*n* = 14)			Group 2 (*n* = 9)		
		T0	T7	*p*-Value	T0	T7	*p*-Value
**Vessel** **density (mm^−1^)**	Central	8.17 ± 3.34	10.27 ±3.94	*p* = 0.016 *	8.25 ± 3.25	12.74 ± 5.89	*p* = 0.022 *
	Inner	16.68 ± 3.04	19.27 ± 2.68	*p* = 0.001 *	15.04 ± 4.37	18.63 ± 2.36	*p* = 0.008 *
	Full	15.70 ± 3.02	18.25 ± 2.93	*p* = 0.01 *	14.26 ± 3.96	17.97± 2.93	*p* = 0.006 *
**Perfusion density (%)**	Central	14.87 ± 6.33	18.22 ±7.11	*p* = 0.03 *	15.06 ± 5.96	22.72 ± 11.11	*p* = 0.039 *
	Inner	31.20 ± 4.98	35.89 ± 4.38	*p* = 0.007 *	28.06± 7.62	35.13 ± 4.04	*p* = 0.006 *
	Full	29.35 ± 5.01	33.75 ± 4.61	*p* = 0.005 *	26.66 ± 6.68	33.73 ± 3.52	*p* = 0.005 *
**FAZ**	Area (mm^2^)	0.27 ± 0.12	0.25 ± 0.10	*p* = 0.05	0.25 ± 0.14	0.22 ± 0.12	*p* = 0.08
	Perimeter (mm)	2.35 ± 0.49	2.23 ± 0.57	*p* = 0.23	2.25 ± 0.63	2.06 ± 0.61	*p* = 0.14
	Circularity Index	0.59 ± 0.06	0.62 ± 0.09	*p* = 0.05	0.60 ± 0.03	0.60 ± 0.09	*p* = 0.9

Abbreviations: FAZ = foveal avascular zone area; mm = millimeter; T0 = baseline values; T7 = 1-week follow-up, *n* = number, * = statistically significant.

**Table 5:** Subgroup analysis of the mean difference [Δ (T7-T1)] between the mean preoperative and postoperative perfusion density, vessel density, and FAZ parameters after cataract surgery according to different stages of cataract severity. FAZ = foveal avascular zone; T0 = baseline values; T7 = 1-week follow-up; Δ = difference.

		Preoperative (T0)			1 Week Postoperatively (T7)			Δ (T7-T1)		
		Group 1 (*n* = 14)	Group 2 (*n* = 9)	*p*-Value	Group 1 (*n* = 14)	Group 2 (*n* = 9)	*p*-Value	Group 1 (*n* = 14)	Group 2 (*n* = 9)	*p*-Value
**Vessel** **density (mm^−1^)**	Central	8.17 ± 3.34	8.25 ± 3.25	*p* = 0.95	10.27 ±3.94	12.74 ± 5.89	*p* = 0.24	2.59 ± 3.33	3.58 ± 3.07	*p* =0.14
	Inner	16.68 ± 3.04	15.04 ± 4.37	*p* = 0.22	19.27 ± 2.68	18.63 ± 2.36	*p* = 0.58	2.63 ± 3.53	3.43 ± 2.83	*p* = 0.48
	Full	15.70 ± 3.02	14.26 ± 3.96	*p* = 0.98	18.25 ± 2.93	17.97± 2.93	*p* = 0.80	2.55 ± 3.18	3.71 ± 3.02	*p* = 0.39
**Perfusion density (%)**	Central	14.87 ± 6.33	15.06 ± 5.96	*p* = 0.52	18.22 ±7.11	22.72 ± 11.11	*p* = 0.24	3.35 ± 5.27	7.65 ± 9.30	*p* = 0.17
	Inner	31.20 ± 4.98	28.06± 7.62	*p* = 0.24	35.89 ± 4.38	35.13 ± 4.04	*p* = 0.88	4.68± 5.37	7.06 ± 5.62	*p* = 0.32
	Full	29.35 ± 5.01	26.66 ± 6.68	*p* = 0.28	33.75 ± 4.61	33.73 ± 3.52	*p* = 0.99	4.40 ± 5.18	7.06± 5.50	*p* = 0.25
**FAZ**	Area (mm^2^)	0.27 ± 0.12	0.25 ± 0.14	*p* = 0.72	0.25 ± 0.10	0.22 ± 0.12	*p* = 0.48	−0.02 ± 0.04	−0.03 ± 0.04	*p* = 0.5
	Perimeter (mm)	2.35 ± 0.49	2.25 ± 0.63	*p* = 0.68	2.23 ± 0.57	2.06 ± 0.61	*p* = 0.51	−0.12 ± 0.36	−0.19 ± 0.35	*p* = 0.65
	Circularity Index	0.59 ± 0.06	0.60 ± 0.03	*p* = 0.97	0.62 ± 0.09	0.60 ± 0.09	*p* = 0.61	0.02 ± 0.13	0.03 ± 0.08	*p* = 0.66

Abbreviations: FAZ = foveal avascular zone area; mm = millimeter; T0 = baseline values; T7 = 1-week follow-up; Δ = differences; *n* = number.

## Data Availability

Data is contained within the article.
